# Noise‐augmented deep denoising: A method to boost CT image denoising networks

**DOI:** 10.1002/mp.18121

**Published:** 2025-09-23

**Authors:** Gernot Kristof, Elias Eulig, Marc Kachelrieß

**Affiliations:** ^1^ Division of X–Ray Imaging and Computed Tomography German Cancer Research Center (DKFZ) Heidelberg Germany; ^2^ Department of Physics and Astronomy Heidelberg University Heidelberg Germany; ^3^ Medical Faculty Heidelberg University Heidelberg Germany

**Keywords:** deep learning, diagnostic CT, dose reduction, neural networks, noise augmentation, noise reduction

## Abstract

**Background:**

Denoising low dose computed tomography (CT) images can have great advantages for the aim of minimizing the radiation risk of the patients, as it can help lower the effective dose to the patient while providing constant image quality. In recent years, deep denoising methods became a popular way to accomplish this task. Conventional deep denoising algorithms, however, cannot handle the correlation between neighboring pixels or voxels very well, because the noise structure in CT is a resultant of the global attenuation properties of the patient and because the receptive field of most denoising approaches is rather small.

**Purpose:**

The purpose of this study is to improve existing denoising networks, by providing them additional information about the image noise.

**Methods:**

We here propose to generate N additional noise realizations by simulation, reconstruct them, and use these noise images as additional input into existing denoising networks. This noise augmentation is intended to guide the denoising process. The additional noise realizations are not only required during training, but also during inference. The rationale behind this noise‐augmented deep denoising (NADD) is that CT image noise is strongly patient‐specific and it is non‐local since it depends on the attenuation of X‐ray beams. NADD is architecture‐agnostic and can thus be used to improve any previously proposed method. We demonstrate NADD using existing denoising networks that we slightly modified in their input layer in order to take the CT image that is to be denoised plus additional noise images as input. To do so, we modified three popular denoising networks, the CNN10, the ResNet, and the WGAN‐VGG and apply them to clinical cases with 90% dose reduction.

**Results:**

In all cases tested, the denoising networks strongly benefit from the noise augmentation. Noise artifacts that are being misinterpreted by the original networks as being anatomical structures, are correctly removed by the NADD version of the same networks. The more noise images are provided, the better the performance.

**Conclusions:**

Providing additional simulated noise realizations helps to significantly improve the performance of CT image denoising networks.

## 1 INTRODUCTION

Computed tomography (CT) is one of the most important imaging modalities in modern medicine. A full‐body scan can be acquired in the matter of seconds, making CT an irreplaceable imaging technique. However, the drawback of using ionizing radiation remains the main concern of CT, as the radiation should be kept as low as reasonably achievable (ALARA principle).^1^, ^2^ Decreasing the exposure to the patient generally reduces the image quality by increasing the noise level. Therefore, it is of great importance to find methods which reduce noise, without the necessity of increasing dose to the patient. In recent years, deep neural networks (DNNs) showed promising results on this task, outperforming conventional denoising algorithms both quantitatively and qualitatively.

Typical neural networks used for noise reduction, such as those in references,^3^, ^4^, ^5^, ^6^, ^7^, ^8^, ^9^, ^10^ for example, have a rather small receptive field, that is, output pixels are influenced by a small corresponding region in the input image. However, image noise in CT is correlated between neighboring voxels and not voxelwise statistically independent, that is, the covariance between voxels is not zero. This correlation depends on the global attenuation properties of the patient and the tube current curve that was used to generate the images. For eccentric cross–sections of the patient, the correlated noise appears as streaks oriented along the direction of highest X‐ray attenuation. For example, consider the shoulder region of a patient where noise appears as streaks oriented along the lateral direction. Due to the small receptive field, the DNNs have not much information about the spatially dependent covariance matrix and about the resulting noise correlation. And even if the receptive field was large enough it may be difficult for the network to leverage the physics behind the CT image formation to conduct the structure of the covariance matrix.

Our aim here is to propose a simple method which implicitly provides such covariance information in image domain and to develop and train neural networks that are able to leverage this information. This is achieved by providing existing DNNs with additional noise realizations for training and inference. In contrast to our previous conference abstract,^11^ we now implement three different neural networks and their noise‐augmented versions, we process far more patient data and we provide quantitative results. Our noise‐augmented deep denoising (NADD) is tested using simulated noise added to clinical CT data. We compare the results to the results of well‐known denoising networks that do not use noise augmentation. All networks are trained using the same training data. Our experiments are done with 2D images, but an extension to 3D is easily possible. There are several other methods addressing the issue of obtaining paired training data for (DNN)‐based denoising schemes, such as in ref.[12, 13, 14]. Those methods use data augmentation or geometrical constraints, in order to achieve denoising in a self‐supervised or unsupervised fashion. In contrast to that, our noise augmentation is used on fully supervised networks in order to enhance their denoising capabilities.

## 2 METHODS

The idea of this project is to generate, by noise injection into the rawdata of the low dose scan followed by image reconstruction, N new noise realizations $h_{n}\in R^{H\times W}$, n=1,…,N of the image $g\left(r\right)\in R^{H\times W}$ that is to be denoised with H and W being the height and width of the reconstructed image, respectively. Here we use up to N=10 noise realizations. These are additional inputs to the DNNs, together with the original low dose CT (LDCT) image that is to be denoised. With these additional input channels the NADD networks can learn about the noise correlation and thereby improve their denoising capabilities.

### 2.1 Noise‐free rawdata

We use a dataset of 39 standard dose, that is, low‐noise, clinical CT volumes, acquired with a Siemens Somatom Force CT system (Siemens Healthineers, Forchheim, Germany). These correspond to approximately 50,000 CT images. Each volume covers the neck to abdomen of the patient. We split the 39 patient volumes as 27:8:4 into training, validation, and test sets. The CT volumes of the patients were forward projected to obtain rawdata p(α,u,v)∈R, with α being the projection angle and u, and v being the two dimensions of the detector, respectively. We will drop the arguments of p whenever they are not necessary and whenever we feel that it is convenient for the reader. We will assume p to be noise‐free.

### 2.2 Noise injection

Given the projection value p, we convert p to intensities as

(1) $$I=I_{0}e^{-p},$$

with $I_{0}\in N$ denoting the number of photons incident on the patient. For convenience we assume $I_{0}$ to be independent of α, u, and v, that is, we did not consider the heel effect, a bowtie filter, tube current modulation, or other effects that would deviate from this assumption.

We use two different values for $I_{0}$. One for the network target (standard dose) and one for the input (low dose), meaning we also inject some small amount of noise into the target projection. This is done so that the noise texture of standard (std) and low dose images is comparable. For convenience, we only describe the noise injection process for the low dose projection, but the noise injection process for the standard dose projection is analogous. We use

(2) $$I_{0}^{\text{low dose}}=\frac{1}{10}I_{0}^{\text{standard dose}},$$

which is the same ratio as in the low dose CT and projection dataset^15^ for chest CT scans.

We now inject Poisson noise by calculating a random variable R such that I+R is Poisson‐distributed. The noise‐injected projection data are now given as

(3) $$\hat{p}\left(\alpha ,u,v\right)=-\ln\left(I(\alpha ,u,v)+R(\alpha ,u,v)\right)/I_{0}.$$

We denote the whole noise injection process as Q(p). This means, we have $\hat{p}\left(\alpha ,u,v\right)=Q\left(p\left(\alpha ,u,v\right)\right)$.

### 2.3 Noisy rawdata and additional input channels

The noisy rawdata that we later want to denoise in image domain are computed as

q=Q(p),q∈R,

that is, by injecting noise into the simulated (nearly) noise‐free rawdata that we had generated by forward projection. From q we generate N noise‐only realizations as

$$r_{n}=Q\left(q\right)-q,r_{n}\in R,$$

for n=1,…,N. Reconstructing q yields the image g(r) that we want to denoise with our network. We denote the reconstruction operator as $X^{-1}:R\to R^{H\times W}$, which is the inverse x‐ray transform. We have $g=X^{-1}q$. We reconstruct the projections using the Feldkamp algorithm,^16^, ^17^ as implemented in reference,^18^ with the following reconstruction parameters:

1. flat detector, five to six circle scans per patient taken at different z positions
2. $R_{F}=650$ mm source‐isocenter distance
3. $R_{D}=420$ mm isocenter‐detector distance
4. $N_{\alpha }=1024$ views covering 360
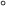

5. $N_{u}=720$ detector columns
6. $N_{v}=256$ detector rows
7. Δu=Δv=1.2 mm detector pixel size
8. $N_{x}=N_{y}=512$ pixels per slice
9. $N_{z}=130,\text{…},150$ reconstructed slices
10. Δx=Δy=Δz=0.5 mm voxel size

Reconstructing the N sinograms $r_{n}$ yields N noise‐only images $h_{n}=X^{-1}r_{n}$ that we use as additional input channels to our networks. From these images NADD shall deduce the required information about the shift‐variant covariances. During training, the data are normalized to have zero mean and unit variance for every input channel.

We chose to use N=10 as the maximum number of additional input channels. We compare the results of each network with those of the conventional network, that is, one that uses N=0 but otherwise has the same structure. We also investigate the impact of the number of channels by choosing different values for N between N=0 and N=10. We will see later that this is a suitable range.

**FIGURE 1:**
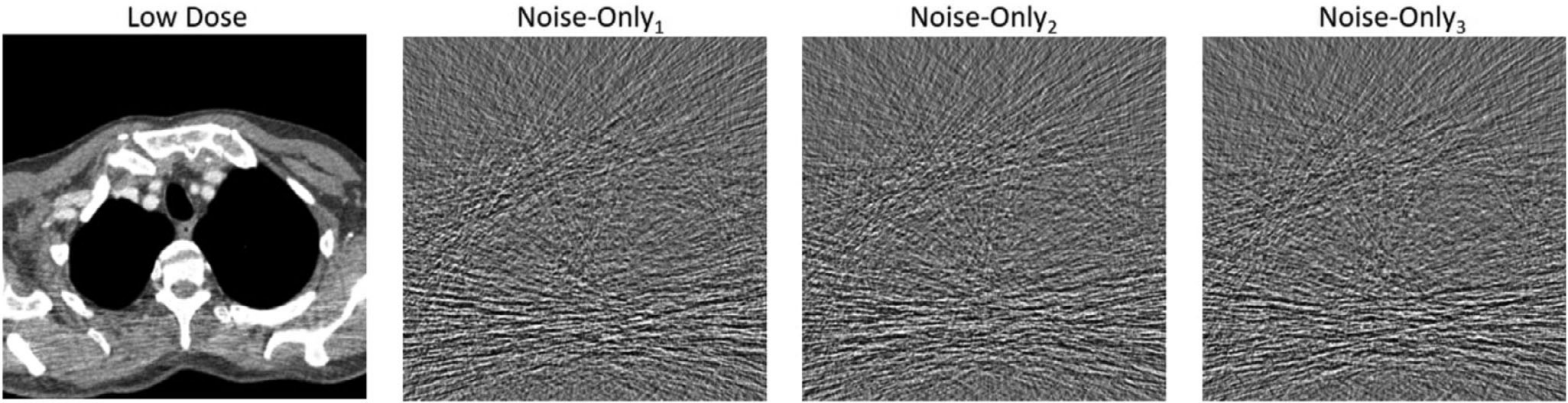
Low dose image g (C=0 HU, W=600 HU) and three corresponding noise‐only images $h_{1}$, $h_{2}$, and $h_{3}$ (C=0 HU, W=150 HU), which are used as additional input in the denoising network.

Figure 1 shows an exemplary low dose image g and three corresponding noise‐only images $h_{1},\text{…},h_{3}$ that would be provided to the denoising network as additional input.

### 2.4 Network architectures

We apply our NADD approach to three popular DNN‐based methods for low dose CT image denoising. The first is CNN10,^3^ which is a simple three layer convolutional neural network (CNN). It aims to minimize the mean squared error (MSE) between its own prediction and the standard dose target. The ResNet^10^ is trained similarly, but employs a deeper network architecture compared to CNN10. The third network we investigate is the WGAN‐VGG,^9^ which is trained in an adversarial fashion.

Every architecture will be trained with and without the additional noise realizations. A subscript will indicate if any additional channels are used and how many. For example ${\mathrm{ResNet}}_{+6}$ would be the ResNet architecture with N=6 additional noise‐only inputs, while CNN10 is the notation for the CNN10 network without noise augmentation.

### 2.5 Evaluation fairness

When evaluating the effectiveness of new methods, it is of great importance to make the evaluation as fair as possible.^19^ For that we will look at two critical reasons, as to why our NADD method could perform better, without actually having a real advantage compared to the standalone DNN architectures.

First, we will address the issues of hyperparameters. Eulig et al.^19^ performed a low dose CT benchmark and have shown the importance of choosing hyperparameters in a fair and consistent way in order to be able to compare different denoising methods. We here use the hyperparameters they found in their automatic hyperparameter optimization for several denoising algorithms.

Secondly, it is obvious, that the noise–augmented versions of the denoising networks have a higher number of trainable parameters than their original versions. This is because the additional input channels increase the number of parameters in the first layer. In order to see whether this leaves our NADD method with an unfair advantage we also generate an adjusted network with higher parameter count. From among the three algorithms tested, CNN10 has the lowest number of parameters and layers. Thus the additional trainable parameters, introduced by NADD, would have the highest impact for this method. CNN10 has convolutional layers with kernel sizes $s_{1}=9$, $s_{2}=3$, and $s_{3}=5$ and $n_{1}=64$ and $n_{2}=32$ features in the first and second layer, respectively. The number of total network parameters of CNN10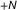, with 1+N input channels, is given by:

(4) $$n_{1}s_{1}^{2}\left(1+N\right)+n_{1}+n_{2}s_{2}^{2}n_{1}+n_{2}+s_{3}^{2}n_{2}+1.$$

This yields 76,353 open parameters for CNN10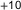. For CNN10 we thus adjust to $n_{1}=205$ and denote the resulting network as CNN10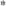. CNN10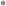 has 76,683 open parameters which is slightly more than the number of parameters of CNN10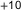. Thus, if it were only the number of network parameters and not the noise augmentation that improves the performance of noise reduction, we would expect CNN10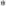 to be on par with or to outperform CNN10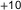. We will see that this is not the case.

## 3 RESULTS

**FIGURE 2:**
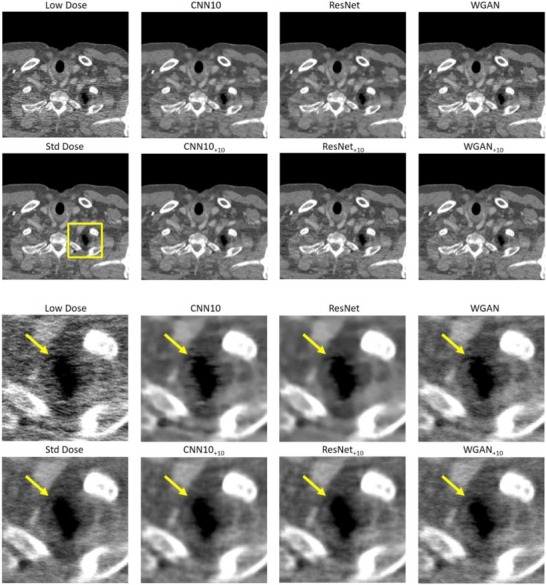
Example slice of an eccentric patient region of Patient 1. The images show strongly correlated noise. Top: Large field of view. Bottom: Zoom‐in. The baseline methods CNN10, ResNet, and WGAN are compared to their NADD versions with ten additional noise realizations. C=50 HU, W=900 HU. CNN, convolutional neural network; NADD, noise‐augmented deep denoising.

**FIGURE 3:**
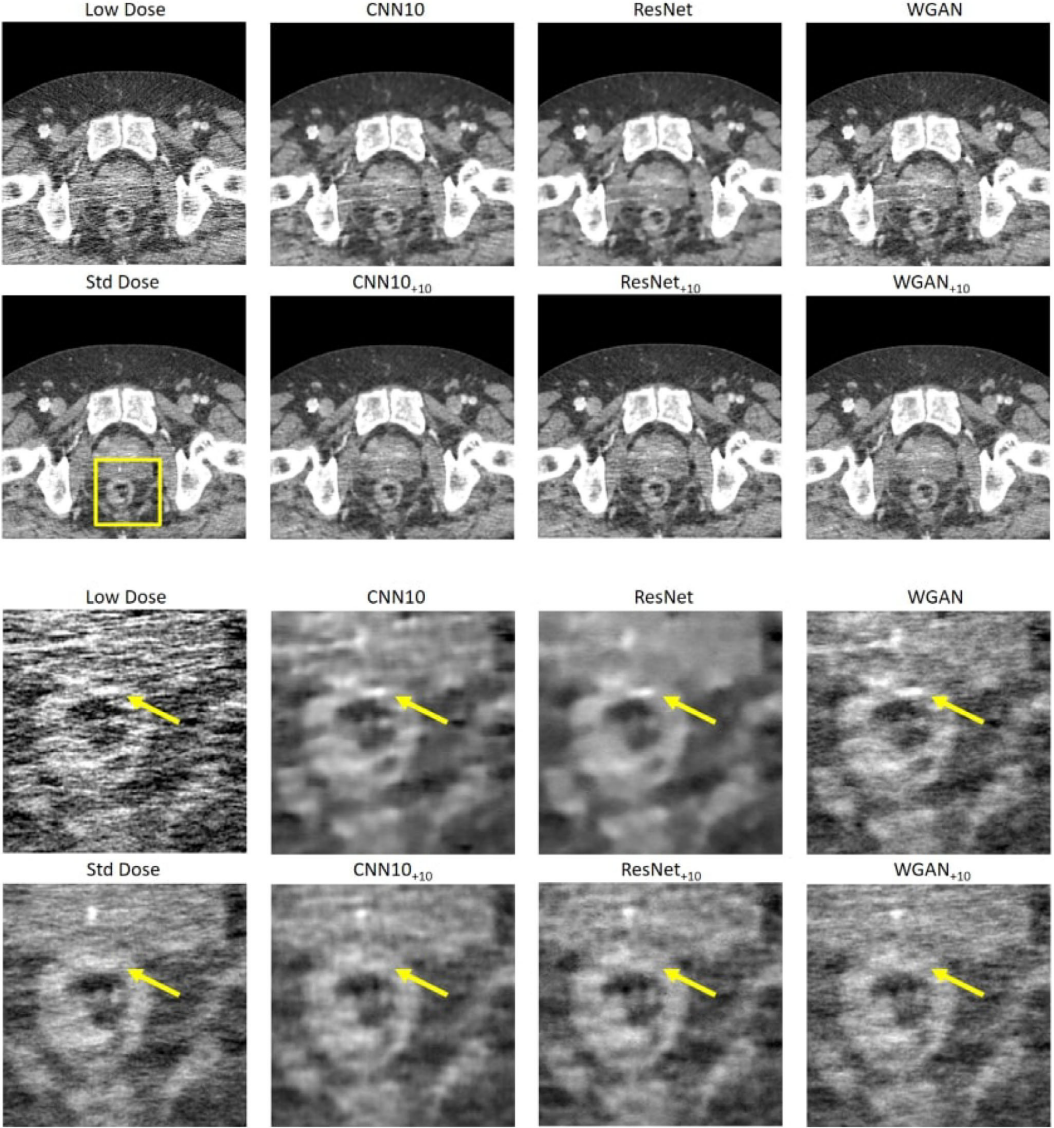
Example slice of an eccentric patient region of Patient 2. Top: large field of view. Bottom: Zoom‐in. The images show strongly correlated noise. The baseline methods CNN10, ResNet, and WGAN are compared to their NADD versions with ten additional noise realizations. C=0 HU, W=500 HU. CNN, convolutional neural network; NADD, noise‐augmented deep denoising.

Figures 2 and 3 show selected slices of two different patients and zoomed‐in regions thereof. We can see the strong correlated noise streaks in the low dose images. These artifacts may impair the reading of the image and could be mistaken for anatomic structures, for example, for lesions. We show the results for each of the three denoising algorithms once without noise augmentation and once the NADD version with ten additional noise‐only images as input. We see that the use of the additional noise input removes certain hyper‐ or hypodense structures introduced by the noise, while the original networks often fail to remove them reliably. In addition, NADD keeps the noise structure closer to the standard dose image, compared to the baseline method.

**FIGURE 4:**
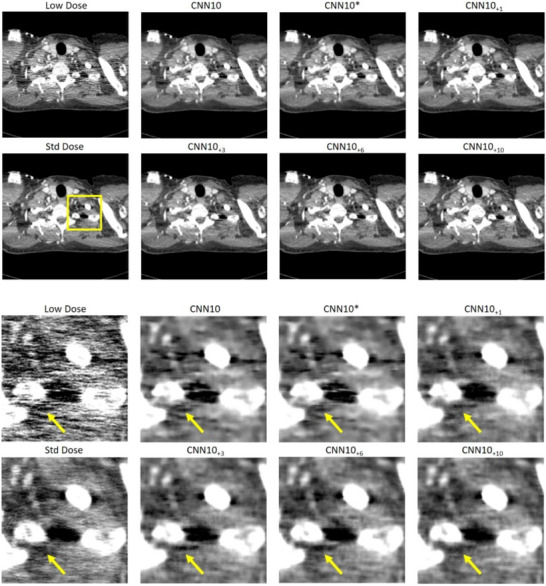
Example slice of an eccentric patient region of Patient 3. Top: Large field of view. Bottom: Zoom‐in. The images show strongly correlated noise. We show the impact of using different values for N in the case of the CNN10 algorithm. The adjusted CNN10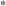 does not improve the denoising capabilities compared to CNN10. C=0 HU, W=600 HU. CNN, convolutional neural network.

**FIGURE 5:**
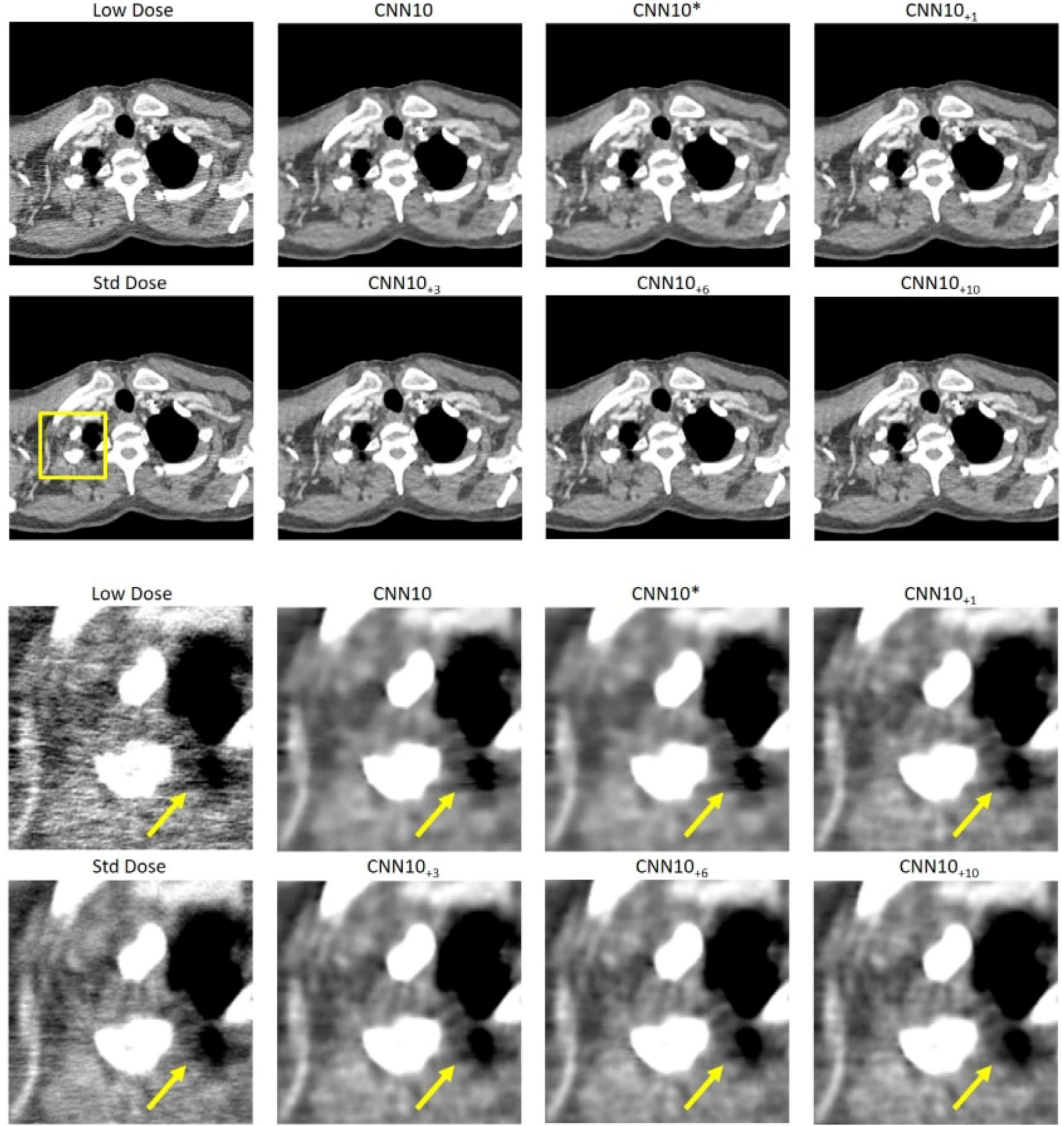
Example slice of an eccentric patient region of Patient 4. Top: Large field of view. Bottom: Zoom‐in. The images show strongly correlated noise. We show the impact of using different values for N in the case of the CNN10 algorithm. The adjusted CNN10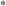 does not improve the denoising capabilities compared to CNN10. C=0 HU, W=600 HU. CNN, convolutional neural network.

In Figures 4 and 5 we show the impact of using a different number N of noise‐only realizations for the CNN10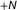 algorithm. We also show results for the CNN10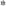 network, which has an adjusted number of trainable parameters. It is apparent that the additional parameters have little impact compared to the impact that noise augmentation has. With NADD, the denoising capabilities increase with an increasing number of denoising realizations. Notably, N=3 noise realizations already show great improvement, compared to the baseline CNN10.

So far, we intentionally selected the images to be eccentric patient cross‐sections. The reasoning for this is that for these cross‐sections the noise becomes strongly correlated, appearing as hyper‐ or hypodense streaks in the image. We can see that those streaks, as well as the overall texture of the image, are better corrected by our method including the additional noise realizations compared to the baseline methods.

**FIGURE 6:**
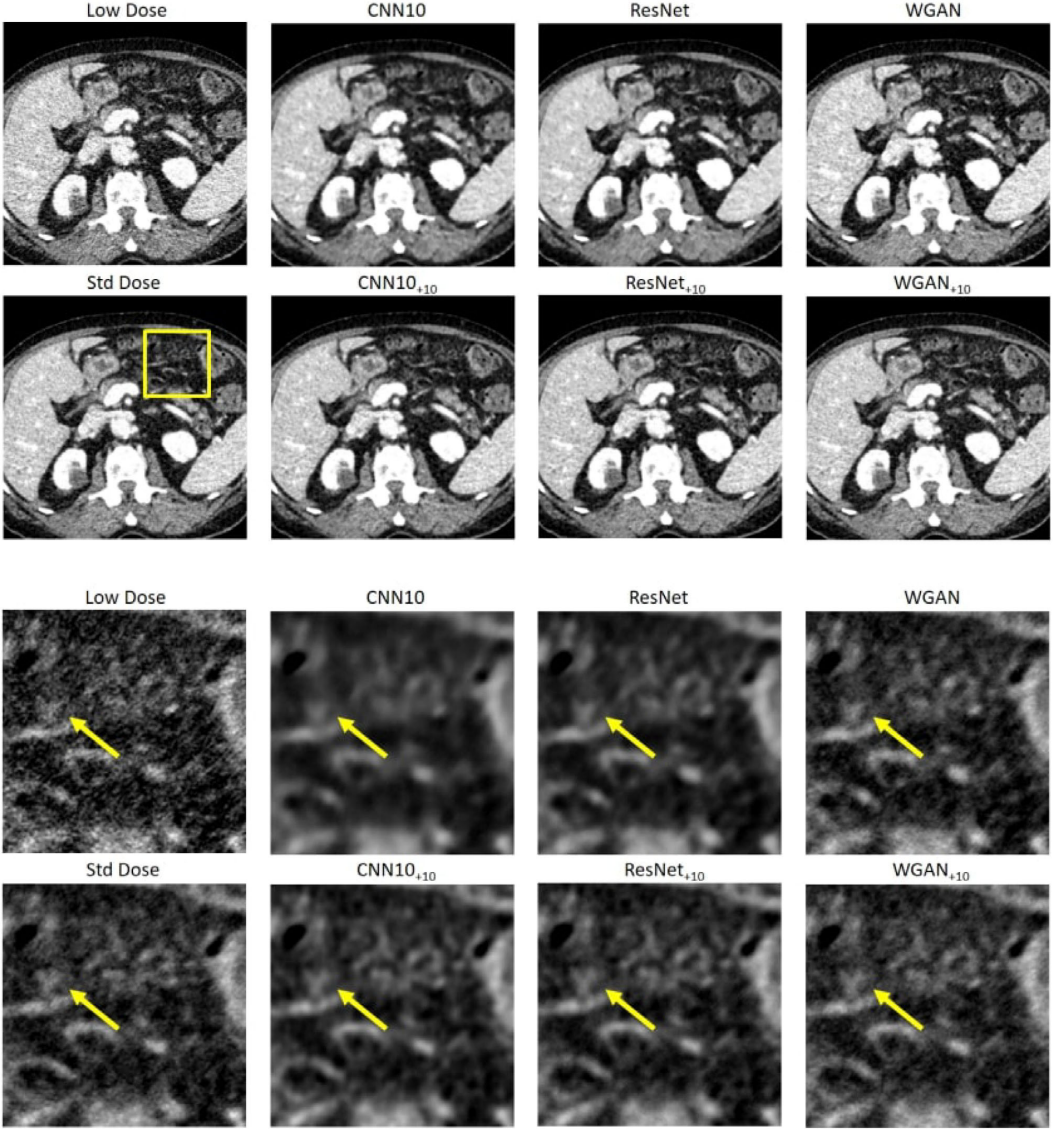
Example slice of a non‐eccentric patient region of Patient 4. Top: Large field of view. Bottom: Zoom‐in. C=40 HU, W=400 HU.

In Figure 6 we show a non‐eccentric region for Patient 4. As expected, the advantages of our method are less obvious, due to the lack of strong correlated noise streaks. A closer look at the NADD images still reveals improvements over the non‐augmented networks.

Table 1 shows the structural similarity index measure (SSIM)^20^ and peak signal‐to‐noise ratio (PSNR), which are two common quantitative measures for image quality assessment. Additionally, we show the visual information fidelity (VIF),^21^ which has been shown to have a higher correlation with human reader studies compared to SSIM or PSNR.^22^, ^23^ We calculated these measures for each slice of the four test patient volumes and averaged over all 5262 slices. We can see, that for all three methods SSIM, PSNR, and VIF are higher with the noise augmentation, compared to the baseline method. Also, all measures increase with increasing number of additional noise realizations N for the CNN10 algorithm.

**TABLE 1:** Average SSIM, PSNR, and VIF results for all test patient slices.

Methods	SSIM	PSNR	VIF
Low dose	0.8494	37.4336	0.1824
CNN10	0.9373	41.6372	0.2615
CNN10 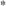	0.9386	41.7871	0.2643
CNN10 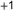	0.9446	42.1535	0.2743
CNN10 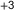	0.9513	42.7117	0.2927
CNN10 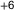	0.9548	43.0454	0.3032
CNN10 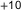	0.9560	43.1015	0.3059
ResNet	0.9417	41.9842	0.2711
${\mathrm{ResNet}}_{+10}$	0.9538	43.0339	0.3047
WGAN	0.9173	40.1954	0.2219
${\mathrm{WGAN}}_{+10}$	0.9404	41.6927	0.2720

*Note*: We see, that for all three denoising algorithms, SSIM, PSNR, and VIF improve with the use of additional noise realizations. For CNN10 we also see that more noise realizations are beneficial.

Abbreviations: CNN, convolutional neural network; PSNR, peak signal‐to‐noise ratio; SSIM, structural similarity index measure; VIF, visual information fidelity.

Each denoising algorithm with N=10 performs significantly better than their traditional counterpart. This was tested on each metric using a one‐sided Wilcoxon signed‐rank test^24^ with significance level α=1%.

**FIGURE 7:**
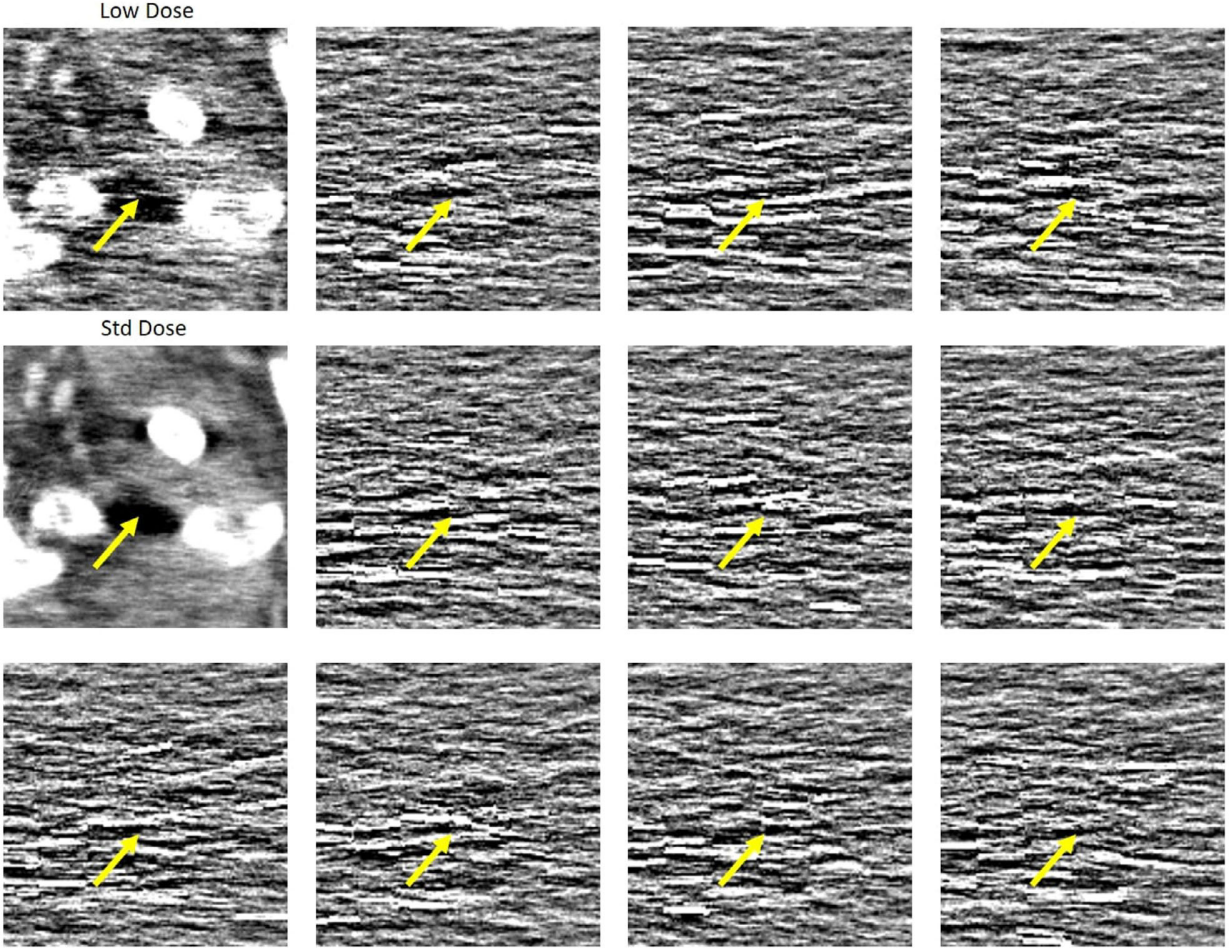
Zoomed‐in low and standard dose region of Patient 3. C=0 HU, W=600 HU. Additionally we show the ten noise realizations corresponding to the slice. The arrows indicate a region where the low dose image shows a streak artifact. We can see the strong streaky structures in the noise images. We hypothesize informing the network about this structure may increase the denoising performance. C=0 HU, W=550 HU.

In Figure 7 we show the low and standard dose slice of Patient 3 and the respective noise realizations of the same slice. Figure 4 shows the improvement of denoising quality by using the additional noise realizations as input. We can see how the noise creates strong streaky artifacts in the low dose image and we can see a multitude of those streaks in the noise images. This indicates that the network does indeed learn to remove these correlated streaks, by having these additional images given as input.

**FIGURE 8:**
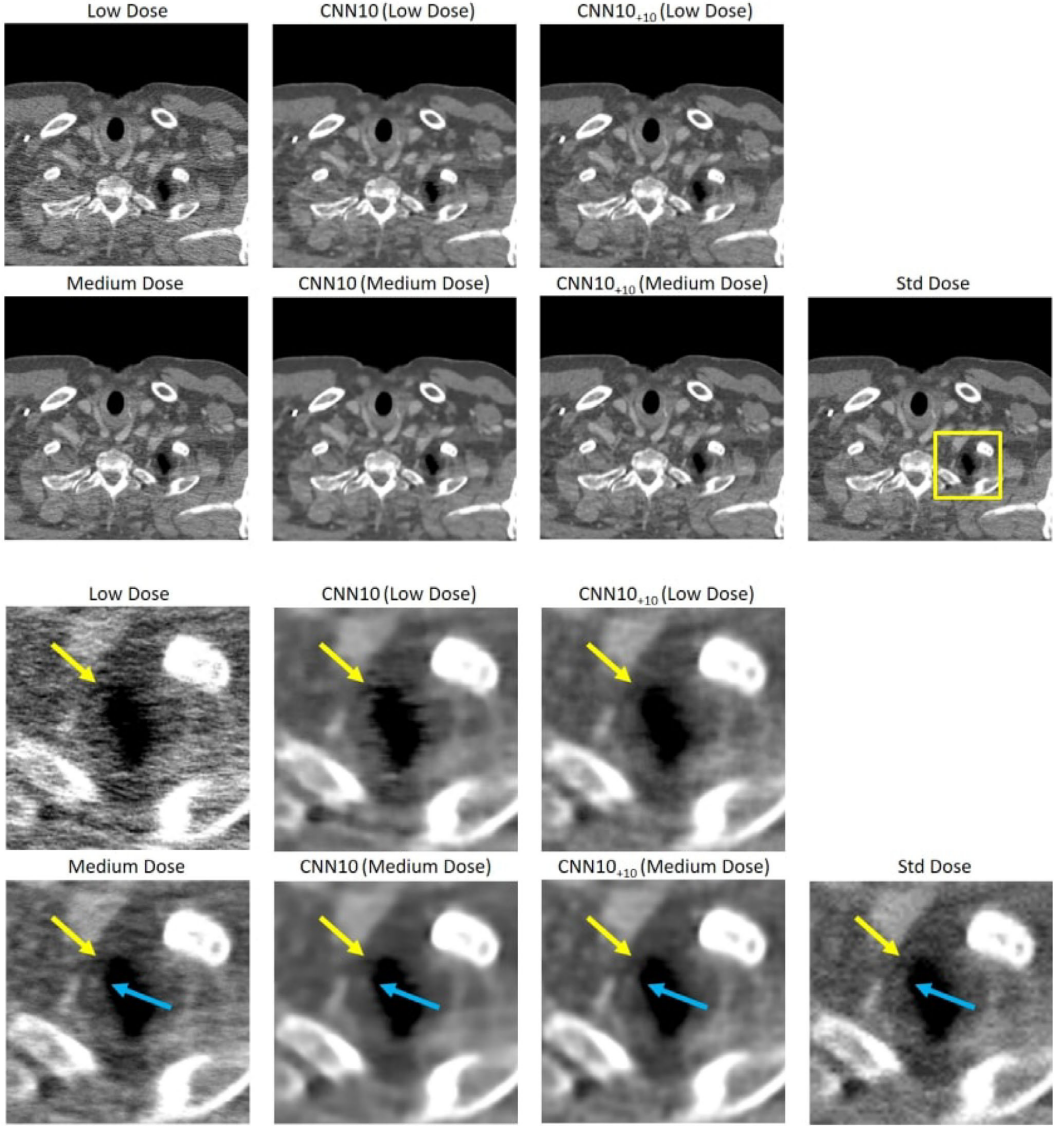
Example slice of an eccentric patient region of Patient 1. Top: Large field of view. Bottom: Zoom‐in. Here we show CNN10 with and without noise augmentation applied to a low dose and a medium dose image. The additional noise realizations required for NADD were calculated at low dose in all cases. C=50 HU, W=900 HU. CNN, convolutional neural network; NADD, noise‐augmented deep denoising.

We evaluated whether the trained networks also work on images with less noise. Therefore we generated images at 25% dose instead of 10%. We will call these 25% dose images medium dose images. We then applied the same networks (no new training) to the medium dose images. The additional noise realizations were calculated based on the medium dose rawdata, however the intensity of the noise‐only realizations were kept at low dose level, which creates a discrepancy between the noise levels of the medium dose and noise‐only images. For all architectures the SSIM, PSNR, and VIF of the medium dose results were higher than the low dose results. This is expected, as the medium dose images are much less noisy. Table 2 shows that the NADD‐enhanced networks still perform significantly better, compared to the baseline methods, although the networks were applied to images with a lower noise level than the one that had been seen during training. This indicates that the method is robust, with respect to the noise level used for the noise augmentation. Figure 8 shows the result for the CNN10 architecture applied to the low and medium dose images. The blue and yellow arrows indicate hyperdense and hypodense structures, respectively, which are introduced by noise. NADD manages to remove these noise‐induced artifacts more reliably than the baseline method in both low and medium dose cases.

**TABLE 2:** Average SSIM, PSNR, and VIF results for all test patient slices at medium dose (left) and at low dose (right).

Methods	SSIM	PSNR	VIF	Methods	SSIM	PSNR	VIF
Medium Dose	0.9459	43.2466	0.3038	Low Dose	0.8494	37.4336	0.1824
CNN10	0.9570	43.4975	0.3201	CNN10	0.9373	41.6372	0.2615
CNN10 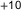	0.9742	45.5090	0.3697	CNN10 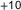	0.9560	43.1015	0.3059
ResNet	0.9618	44.0108	0.3326	ResNet	0.9417	41.9842	0.2711
${\mathrm{ResNet}}_{+10}$	0.9742	45.8137	0.3795	${\mathrm{ResNet}}_{+10}$	0.9538	43.0339	0.3047
WGAN	0.9555	43.0374	0.2950	WGAN	0.9173	40.1954	0.2219
${\mathrm{WGAN}}_{+10}$	0.9682	44.6206	0.3488	${\mathrm{WGAN}}_{+10}$	0.9404	41.6927	0.2720

*Note*: For inference we used the low dose noise‐only realizations for both, the medium and the low dose cases. For all denoising algorithms, SSIM, PSNR, and VIF improve with the use of additional noise realizations. The low dose values were taken from Table 1.

Abbreviations: CNN, convolutional neural network; PSNR, peak signal‐to‐noise ratio; SSIM, structural similarity index measure; VIF, visual information fidelity.

## 4 DISCUSSION

In this study, we propose to use noise‐augmented input for denoising neural networks. This input also needs to be provided during inference since the noise correlations in CT are patient‐specific. We here do not introduce new denoising networks. Rather, we use existing networks that we slightly modified to allow for the additional input channels.

We showed that using such additional noise‐only realizations can significantly improve the denoising capabilities of denoising neural networks. The additional information about noise correlations enables the networks to reduce undesired noise streaks and to produce a more realistic noise texture in the denoised image. This improvement can be seen among the three different denoising algorithms, that we tested, CNN10, ResNet, and WGAN, with the latter being trained in an adversarial fashion.

The calculated SSIM, PSNR, and VIF measures quantitatively confirm our findings. Notably, when looking at the measures of the four test patients separately, for one of the four patients the PSNR was slightly higher for CNN10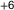 compared to ${\mathrm{CNN}}_{+10}$. This indicates that our tested range between N=0 and N=10 makes some sense and going much further than 10 will not yield great additional advantages.

This study is a proof of concept and has the following limitations. For simplicity, we limited the networks to two dimensions. The improvement may increase for three‐dimensional denoising with three‐dimensional noise input. Also, the low dose projection noise is simulated and not measured. Future work should include using measured noise for the low dose image, with a more realistic noise texture, and to find out whether the network must be retrained for different reconstruction parameters. Clinical CT data will not have significantly different noise profiles than our simulations. They do have cross‐talk between detector elements and they do have electronic noise. Vendors know their noise quite well and hence can simulate it accurately, which enables them to simulate the additional noise realizations needed for NADD. Such noise‐injection software already exists. An example is the ReconCT software from Siemens, which can accurately inject and simulate the noise of the scanner.^25^, ^26^ Furthermore, we note that the best evaluation method would be to conduct human reader studies.^27^ Evaluation of our method via human reader studies is out of the scope of this work and left for future investigations. Lastly, future studies should investigate whether networks trained with NADD are less invariant to anatomical structures^28^ and less likely to hallucinate,^29^, ^30^ compared to their traditional counterparts.

Notably, NADD would also work for normal dose scans. However, the results may be a lot less impressive, because noise is not the dominant factor in image quality for such images, and therefore noise reduction is less important.

A downside of NADD is the requirement of N additional image reconstructions for inference, as well as a slightly increased inference time for the network due to the additional input.

In conclusion, noise‐augmented deep denoising has the potential to significantly outperform the baseline denoising techniques due to the added knowledge of the noise texture and the covariances.

## CONFLICT OF INTEREST STATEMENT

The authors declare no conflicts of interest.
